# Rosetta Stone for Cancer Cure: Comparison of the Anticancer Capacity of Endogenous Estrogens, Synthetic Estrogens and Antiestrogens

**DOI:** 10.3389/or.2023.10708

**Published:** 2023-04-19

**Authors:** Zsuzsanna Suba

**Affiliations:** Department of Molecular Pathology, National Institute of Oncology, Budapest, Hungary

**Keywords:** aromatase inhibitor, DNA damage, DNA repair, endocrine disruptor, estrogen receptor, growth factor receptor, mutation, tamoxifen

## Abstract

This work presents the history of the recognition of principal regulatory capacities of estrogen hormones having been mistakenly regarded as breast cancer promoting agents for more than 120 years. Comprehensive analysis of the results of clinical, epidemiological, immunological and molecular studies justified that endogenous estrogens are the principal regulators of embryonic development, survival and reproduction *via* orchestrating appropriate expression and even edition of all genes in mammalians. Medical use of chemically modified synthetic estrogens caused toxic complications; thromboembolic events and increased cancer risk in female organs as they proved to be endocrine disruptors deregulating estrogen receptors (ERs) rather than their activators. Synthetic estrogen treatment exhibits ambiguous correlations with cancer risk at different sites, which may be attributed to an inhibition of the unliganded activation of estrogen receptors (ERs) coupled with compensatory liganded activation. The principle of estrogen induced breast cancer led to the introduction of antiestrogen therapies against this tumor; inhibition of the liganded activation of estrogen receptors and aromatase enzyme activity. The initial enthusiasm turned into disappointment as the majority of breast cancers proved to be primarily resistant to antiestrogens. In addition, nearly all patients showing earlier good tumor responses to endocrine therapy, later experienced secondary resistance leading to metastatic disease and fatal outcome. Studying the molecular events in tumors responsive and unresponsive to antiestrogen therapy, it was illuminated that a complete inhibition of liganded ER activation stimulates the growth of cancers, while a successful compensatory upregulation of estrogen signal may achieve DNA restoration, tumor regression and patient’s survival. Recognition of the principal role of endogenous estrogens in gene expression, gene edition and DNA repair, estrogen treatment and stimulation of ER expression in patients may bring about a great turn in medical practice.

## Introduction

The role of estrogen hormones in the carcinogenesis of female breasts has long been debated based on the ambiguous results of menopausal hormone therapy (MHT), however, the exact mechanism of their tumor inducing effect is not entirely clarified till now (1).

In 1896, a transient tumor regression was experienced in the minority of premenopausal breast cancer cases *via* estrogen withdrawal by oophorectomy (2). From that time onwards, hypoestrogenism gained great popularity in the medical practice of breast cancer care; however, recently the use of aromatase inhibitors has become a less invasive means of estrogen withdrawal (3). The shock of drastic estrogen withdrawal by oophorectomy may result in a deceiving transient tumor regression in certain breast cancer cases *via* a quick compensatory estrogen synthesis at the extragonadal sites. Nevertheless, mutilating surgical therapy could not achieve a reassuring advance in breast cancer care (4).

In the early 1940s, the US Food and Drug Administration (FDA) approved marketing of synthetic estrogens, non-steroidal diethylstilbestrol (DES) and steroidal ethinylestradiol (EE) as well as conjugated equine estrogens (CEEs) for medical purposes (5).

In 1944, Haddow et al. proposed the concept of “the estrogen paradox” suggesting that in spite of the well known stimulating effect of estrogen hormones on breast cancer, high doses of DES is a promising therapy against this tumor (6). Despite the experienced low tumor response rates (<30%) and serious toxic side effects, the use of high dose synthetic estrogen therapy turned into the standard of care for postmenopausal breast cancer patients (7). CEE in high doses was not applied for breast cancer therapy because of its natural hormone derivative content.

In the meantime, the controversial results of great menopausal hormone therapy (MHT) studies strengthened the presumed role of estrogen treatment in the development and progression of breast cancer (8, 9). Among postmenopausal women, the use of exogenous estrogens having synthetic or natural origin and their combinations with synthetic progestins resulted in ambiguous clinical experiences. MHT yielded unforeseeable risks and benefits concerning arterial and venous thromboembolism and cancers of breasts and endometrium. According to the guidance of Food and Drug Administration (FDA), the benefits of MHT surpass their risks, while there were no comparative informations regarding the efficacy and toxicity of bioidentical versus conventional hormones (5).

Separated evaluation of the effects of specific HRT types applying natural and synthetic hormones justified that horse urine derived CEE alone is an outstanding formula decreasing the risk for breast cancer, coronary heart disease and bone loss. Conversely, synthetic estrogens and their combination with synthetic progestins may have unforeseeable toxic and carcinogenic impacts (10).

The presumed carcinogenic capacity of endogenous estrogens promoted the introduction of current antiestrogen therapies against breast cancer. In 1971, Cole et al. reported the use of tamoxifen, a selective inhibitor of liganded activation of estrogen receptor (ER) for the treatment of advanced breast cancer in postmenopausal women (11). The experienced tumor response rates were similarly low in patients treated with either tamoxifen or DES (<30%), however, toxic side effects experienced in tamoxifen treated patients proved to be less dramatic (12). Tamoxifen became a preferred first line therapy for postmenopausal breast cancer cases and it completely replaced the use of high dose synthetic estrogen treatment (7).

Antiestrogen therapy of advanced breast cancer yielded many difficulties and failures from the onset. Tamoxifen as a first line therapy induced moderate tumor regression rate (<40%–50%) even among the targeted ER-positive breast cancer cases, while the remaining patients could not exhibit tumor responses or experienced tumor growth. Therapeutic failures were designated as *de novo* (primary) antiestrogen resistance (13). During long term tamoxifen administration, near all earlier responsive breast cancers exhibited an “acquired (secondary) antiestrogen resistance” resulting in rapid progression of the disease (14). In addition, tamoxifen treatment induced various toxic side effects, which could occasionally be life threatening, such as stroke, coronary heart disease, pulmonary emboli and malignancies at various sites particularly in the endometrium (15).

Aromatase inhibitors (AIs) also were developed for the therapeutic reduction of estrogen synthesis in breast cancer cases (16). Among AI treated patients, the experienced tumor response rate also was low (<30%) similarly to the results of other endocrine therapies. AI treatment against breast cancer seemed to be somewhat safer than tamoxifen use; it provoked somewhat lower rates of thromboembolic complications and endometrial toxicity. *De novo* or acquired resistance to AI treatment also developed in the vast majority of patients with advanced breast cancer.

Following the failures in high dose estrogen treatment the development of antiestrogen therapy could also not realize the enthusiastic expectations for breast cancer defeat. In breast cancer cases diagnosed and treated at the earliest stage, unforeseeable tumor recurrence and fatal outcome may occur even after decades (17). Tumor responses to antiestrogen treatment were transient and inconsistent coupled with high toxicity in breast cancer cases (18). These experiences strongly suggest that our therapeutic efforts against breast cancer are not appropriate. Further insights into the mechanisms of tumor growth and tumor recurrence are necessary for the improvement of breast cancer care.

The comparison between the major anticancer capacity of endogenous estrogens and the ambiguous effects of the endocrine disruptor synthetic estrogens and antiestrogens shows some parallelism with the history of Rosetta stone. It is a stele composed of granodiorite inscribed with three versions of a decree issued in Memphis, Egypt, in 196. The texts are in ancient Egyptian hieroglyphic scripts, in Demotic scripts and in Ancient Greek. The comparison of the three versions made Rosetta stone a key to deciphering the ancient Egyptian scripts.^1^

## Estrogen Deficiency and ER Resistance Were Recognized as Cancer Risk Factors

In 2007, estrogen deficiency was exposed as a newly recognized risk factor for oral cancer in a Hungarian clinical-epidemiological study (19). Oral cancer exhibited increasing prevalence with ageing, while premenopausal women with healthy cycles were strongly protected from this disease compared to age matched men. The male-female ratio of overall oral cancer cases was 3.8:1 showing a high male predominance. Examining the male-female ration of oral cancer cases in the different age groups, it was the highest between 35–40 years of age; 81% versus 19%. Above 50, the percentage of female patients showed a slight increase, while above 70 a steeper increase was observed till 80, when the oral cancer incidence was equalized between male and female patients: 1:1. These data suggested that with ageing, women lose their hormonal protection against oral cancer attributed to their deepening postmenopausal estrogen loss and the associated changes in gene regulation (20). The significantly high rate of early menopause and premenopausal hysterectomy in the anamnesis of elderly female oral cancer cases also supported that estrogen deficiency is a causal factor of this tumor.

Oral cancer induced by decreased estrogen levels seemed to be highly controversial to the old principle of estrogen induced breast cancer as the regulation of genomic processes may not be quite different at various sites. Analyzing the literary data, defective estrogen signaling proved to be a strong cancer risk factor for several organs including female breasts (20). The correlation between premenopausal hormonal defects and the increased risk for gynaecologic and breast malignancies justified that organs with high estrogen need are particularly endangered by the defect of estrogen signal (21).

Estrogen deficiency emerged as a new cancer risk factor directly deteriorating the signaling functions of mammalian cells. Moreover, results of clinical and experimental studies justified that behind insulin resistance associated chronic diseases a defect of estrogen signal may always be revealed suggesting their strong partnership in cancer development (22).

Studies on breast cancer epidemiology suggested that the better the reproductive capacity of women the lower is their breast cancer risk. In healthy premenopausal women having higher estrogen levels, the breast cancer risk is much lower as compared with postmenopausal cases showing extremely low serum estrogen concentrations (23). Studies examining the serum estrogen levels in young and postmenopausal breast cancer cases, could not find direct correlations between increased serum estrogen concentrations and breast cancer development (24, 25). Parity and multiparity in particular, are associated with strikingly decreased breast cancer risk (26). Conversely, anovulatory disorders and nulliparity are well-known risks for breast tumors and further female cancers (27, 28).

Immunohistochemical imaging of ERs, progesterone receptors (PRs) and human epidermal growth factor receptors (HER2s) in breast cancers provided further possibilities to clarify the risk factors and therapeutic possibilities of variously differentiated tumors (29).

Experimental and clinical studies examined the correlation between ER expression and the development and progression of breast cancer. The differences between ER-positive and ER-negative tumors regarding their gene expressions and genomic mutations remained to be clarified (30). However, ER-positive status in tumors is associated with more differentiated and less invasive tumors, suggesting that ERs may have rather a protective role against tumor growth and metastatic progression. In addition, ER-negative and even triple receptor negative tumors (TNBCs) with lack of ER, PR and HER2 expression exhibit poorly differentiated morphology and highly aggressive clinical course. The risk of these aggressive breast cancers is increased in patients with defective estrogen signaling including obesity, metabolic syndrome, type-2 diabetes, breast cancer (BRCA) gene mutation and low environmental light exposure (31). These observations and the high frequency of tumor resistance to antiestrogenic treatment suggest that estrogens and their receptors may have apoptotic action on breast cancer cells rather than increasing their proliferative activity.

In postmenopausal women with decreased estrogen levels, the majority of tumors proved to be highly differentiated with abundant ER expression (32) showing a compensatory reaction in the estrogen deficient milieu. Conversely, premenopausal breast cancer cases with preserved or even increased ovarian estrogen synthesis exhibit a higher rate of ER-negative and even triple negative breast cancers (TNBCs) (33). In these young cases, hyperestrogenism coupled with clinical signs of estrogen deficiency suggest baseline serious defects of ER expression/activation in the background as initiators of poorly differentiated tumors (31).

In animal experiments, high estrogen concentrations in pregnancy and short term exposure to pregnancy mimicking high estrogen levels prevented mammary carcinogenesis induced by either chemicals or tumor inoculation (34). Genetic studies have demonstrated that ovarian estrogens exhibit strong interplay with p53 tumor suppressor protein in rendering the mammary epithelium resistant to carcinogenesis. In addition, estrogen mediated genomic protection in mammary tissue was observed even in mice with homozygous deletions in the gene of p53 suggesting that estrogen treatment is a potent inhibitor of tumorgenesis through multiple pathways (35). Analysis of the failures of currently applied treatments for breast cancer, exogenous estrogen was suggested as a causal therapy against this disease (36).

In conclusion, either estrogen deficiency or ER-resistance to estrogen activation may lead to genomic disorders increasing the risk of breast cancer (37). Moreover, the stronger the defect of estrogen signal, the higher is the risk for poorly differentiated ER-negative and TNBC type tumors.

## Estrogen Activated ERs are Principal Regulators of All Genomic Processes Providing Surveillance for Somatic and Reproductive Health in Mammalians

The genome of living organisms is inherited; however, it shows a great plasticity through appropriate self-mediated changes continuously updating the regulatory processes throughout the life. Bio-intelligence means that the genome of living creatures is capable of perception of its environment (outer and inner) (38). Genome has memory for evaluation of the observed changes, and can decide and carry out mutational responses so as to improve the possibilities for survival and reproduction.

Malignant tumor cells are mistakenly regarded as enemies to be killed similarly like exogenous microbes. Presumably, “hostile” tumor cells develop survival techniques so as to escape from the regulatory commands of patient’s body. In reality, tumor cells are dysregulated human cells having more or less remnants of the same regulatory network like patients’ healthy cells have. The tumor initiator is an inherited or acquired failure of the principal regulator of the genomic machinery. Variously differentiated tumor cells exhibit spontaneous efforts for the improvement of their genomic regulation; however, this work in itself may rarely lead to a successful tumor regression. Tumor cells need medical help for the self directed repair of genomic alterations instead of crude inhibition of their spontaneous gene repairing efforts (39).

Over the past decades, we acquired a deeper appreciation on the roles of estrogen receptors in human physiology and pathology (40). Estrogen activated ERs act as hubs in the network of transcriptional and translational genomic processes. ERs were favored by evolution as being integrators between reproductive and somatic functions. ERs (ER-alpha and ER-beta) are transcriptional factor proteins. In estrogen activated form, they are capable of occupation of all human genes inducing gene expression, ribonucleic acid (RNA) transcription and translational protein synthesis, orchestrating the work of whole genomic machinery.

In humans, ER-alpha and ER-beta isoforms are co-expressed in many cells and tissues, and they control key physiological functions in all organ systems in strong interplay. ER alpha primarily drives and controls the proliferation and DNA stability of cells, while ER beta regulates cell growth in particular (41). Both ER isoforms are mandatory regulators of cellular glucose uptake since all ER driven genomic processes require an appropriate supply of fuel for metabolic processes (42, 43).

ERα is the highly predominant isoform in mammary gland, uterus, ovary (thecal cells), bone, male reproductive organs (testes and epididymis), prostatic stroma, liver, and adipose tissue. By contrast, ERβ is highly expressed in the prostatic epithelium, bladder, ovary (granulosa cells), colon, adipose tissue, and immune system. Both subtypes are markedly expressed in the cardiovascular and central nervous systems (44). This latter experience may explain the high vulnerability to even mild defects of estrogen signal in the heart, arteries and brain. Strict balance of the two receptor isoforms at different sites may be responsible for a fine tuning of regulation affecting the individual development, growth and function of different organs.

ERs have possibilities for liganded (estrogen bound) activation through their activation function 2 (AF2) domain and for unliganded activation by several transduction molecules through the ancient activation function 1 (AF1) domain (40). In the fetal life, differentiation is predominantly driven by the ancient unliganded ER activation. In rapidly growing children, the primacy of growth factors favors unliganded ER activation; while in the adult, reproductive life period, estrogen activation of ERs has a priority in both men and women. In estrogen deficient periods, increased growth factor receptor (GFR) expression and activation may transiently maintain the appropriate ER activation *via* unliganded pathway (45). Ligand-independent responses are ER-mediated effects seen after activating other pathways, such as insulin like growth factor IGF1 receptor–mediated signaling, that results in ER-mediated transcriptional responses independent of estrogenic steroid ligands (46). Balanced liganded and unliganded activation of ERs stimulates estrogen synthesis and ER expression ensuring DNA stabilization and upregulation of the whole genomic machinery (47). Artificial inhibition of either liganded or unliganded activation of ERs induces a strong compensatory upregulation of the unaffected domain, while the failure of restorative efforts may eventually lead to a breakdown of the whole regulation.

## Regulatory Circuits of Activated ERs

**FIGURE 1:**
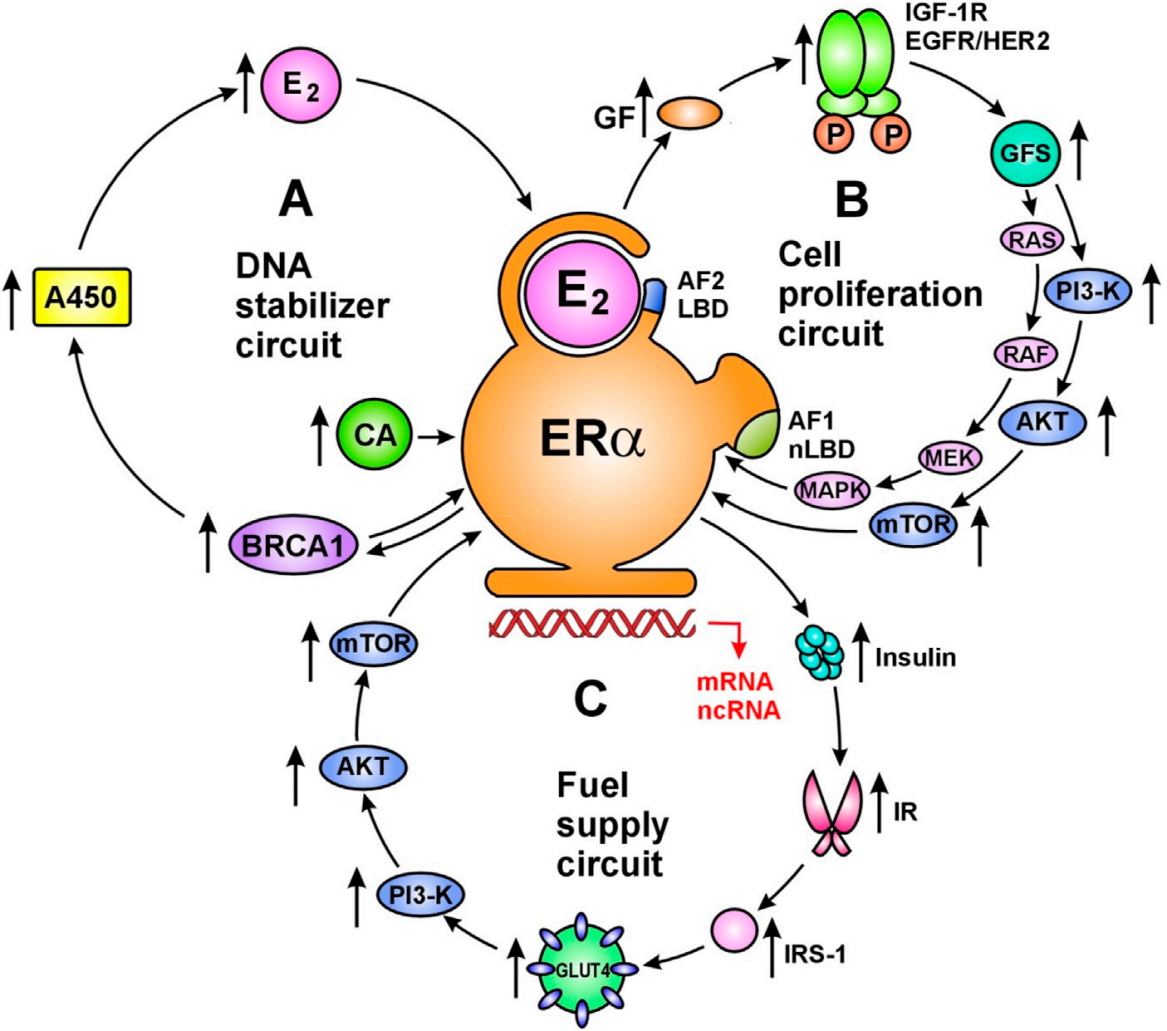
Main regulatory circuits of liganded ER-alpha for DNA stabilization **(A)**, cell proliferation **(B)** and fuel supply **(C)**. Circuit of DNA stabilization **(A)** Estrogen (E_2_) activated estrogen receptor alpha (ERα) upregulates estrogen signal *via* a regulatory circuit together with genome stabilizer protein (BRCA1) and aromatase enzyme (A450). Activated ER-alpha induces messenger RNA (mRNA) expressions on *ESR1*, *BRCA1* and *Cyp19A* aromatase promoter regions upregulating the synthesis of ER-alpha, BRCA1 protein and aromatase enzyme. Aromatase enzyme produces estrogen hormones for further ER activation. In addition, activated ER-alpha may induce activating mutations on *ESR1*, *BRCA1* and *Cyp19A* genes through the expression and activation of appropriate long non-coding RNAs (lncRNAs). Moreover, ER-alpha and BRCA proteins are capable of direct binding as transcriptional factors regulating each-other’s activity. Circuit of cell proliferation **(B)** Estrogen activated ERα is the crucial regulator of increased and decreased cell proliferation in strong interplay with membrane associated tyrosine kinase growth factor receptors; EGFRs and IGF-1Rs. ERs also regulate the expression and activation of growth factors (GFs) and their receptors. Transduction of growth factor signal (GFS) induces kinase cascades *via* PI3-K/AKT/mTOR and RAS/RAF/MEK/MAPK pathways conferring unliganded activation for nuclear ERs and promoting specific gene expressions. Circuit of fuel supply **(C)** Estrogen activated ERα is the regulator of all steps of cellular glucose uptake and the maintenance of glucose homeostasis. Estrogen regulated genes stimulate both insulin synthesis and insulin receptor (IR) expression. Activated ERα stimulates the expression and translocation of glucose transporter 4 (GLUT4) facilitating cellular glucose uptake. In addition, estrogen activated ERα at the plasma membrane stimulates the kinase cascade of PI3-K/AKT/mTOR pathway *via* insulin receptor substrate 1 (IRS-1) activation. These signals induce specific gene expressions in the nucleus conferred by unliganded ERα activation. Abbreviations: LBD, ligand binding domain; CA, coactivator; AF2, activating function 2; AF1, activating function 1.

Activated ERs drive the network of whole genomic machinery stimulating and silencing all physiological processes *via* regulatory circuits (42). The principally important regulatory circuits serve DNA stabilization, cell proliferation/silencing and cellular fuel supply (Figure 1). In a healthy genome, there is no unrestrained activation or blockade of cellular processes, but rather ERs harmonize all physiological activities *via* upregulative or downregulative circuits.

### Regulatory Circuit of DNA Stabilization

There is a primacy of the DNA stabilizer circuit as unrepaired DNA damages endanger the work of whole genomic machinery and the life of living organisms (42). Estrogen activated ER-alpha increases the expression and activation of genome stabilizer proteins, such as BRCA1/2. BRCA proteins as transcriptional factors are capable of increasing the expression and activation of aromatase enzyme. Increased aromatase activation results in new estrogen synthesis, further increasing the liganded activation of ERs and new ER expression. In conclusion, the ^E2^ER–BRCA–aromatase–E2—ER circuit ensures the continuous maintenance of both genome stabilization and estrogen signal. In pregnancy, strong upregulation of estrogen signal and DNA stabilization ensure the safety growth and development of the fetus. Estrogen loss or ER resistance are emergency situations leading to rapid mobilization of ER expression and estrogen synthesis so as to restore the DNA stabilizer circuit. In tumors, estrogen treatment induces upregulation of the whole genomic machinery promoting self directed apoptotic death in tumor cells (42).

### Regulatory Circuit of Cell Proliferation

In the regulatory circuit of cell proliferation estrogen activated ERs drive and control the growth and involution at all sites of the body in strong interplay with membrane associated tyrosine kinase growth factor receptors: epidermal growth factor receptors (EGFRs) and insulin-like growth factor-1 receptors (IGF-1Rs) (48). Liganded ERs regulate the expression and activation of both growth factors (GFs) and growth factor receptors (GFRs). Transduction of growth factor signal (GFS) induces kinase cascades *via* PI3-K/AKT/mTOR and RAS/RAF/MEK/MAPK pathways conferring further unliganded activation to nuclear ERs (39). In pregnancy, extremely high estrogen level drives the enormous growth of uterus, while a drop of estrogen level orchestrates its postpartum involution (45). The emergency situation of low estrogen level leads to increasing expression/activation of GFRs so as to strengthen the estrogen signal *via* unliganded activation of nuclear ERs. In genomically dysregulated tumors, artificial blockade of estrogen signal activates growth factor kinase cascades sending forewarning messages for nuclear ERs *via* unliganded pathway (39).

### Regulatory Circuit of Fuel Supply

In the *regulatory circuit of fuel supply*, activated ERs drive and control all steps of cellular glucose uptake and the maintenance of glucose homeostasis (49). Appropriate insulin assisted glucose uptake is the prerequisite of all somatic and reproductive cellular functions. Estrogen treatment defends the vitality of pancreatic islet cells from lipid deposition (50), and activates their insulin secretion (51). Estrogen increases insulin stimulated cellular glucose uptake *via* facilitating the expression and translocation of intracellular glucose transporters (GLUTs) (52). Disorders of glucose uptake always reflect the manifestation of a defective estrogen signaling in the background (43). In insulin resistant status, estrogen treatment provides liganded activation to ER-alpha, which stimulates GFR signal and tyrosine kinase cascades conferring even an unliganded activation to nuclear ERs (39). In MCF-7 breast cancer cell lines, 17β-estradiol treatment activated glucose uptake *via* GLUT4 translocation and PI3K/Akt signaling pathway (53). In tumor cells, estrogen induced activation of glucose uptake helps the self directed repair of genomic regulation instead of promoting unrestrained proliferation (42).

ERs expressed in the adipose tissue mass regulate the energy supply of the whole body of mammalians. Abdominally located fatty tissue is a hub of the signaling network providing energy and ER signal to the work of visceral organs. Fatty tissue in the pericardium regulates heart, while fat positioned along great vessels drives and controls the function of arteries and veins (54). Increasing estrogen level in adipocytes activates ERs driving the expression of numerous genes and the synthesis of signaling molecules such as sex steroid hormones, adipokines, growth factors and cytokines (49).

Conversely, estrogen deficiency or ER resistance deregulates the signaling functions of adipocytes. In women, menopause associated estrogen loss may increase the risk for both metabolic disorders and weight gain (55). Adipocytes with excessive lipid deposition lose their regulatory function and become insulin resistant. In obesity, adipose tissue mass exhibits low grade inflammation with abundant macrophages and T-cells. Macrophages produce cytokines generating a compensatory increased activation of aromatase enzyme and amplified estrogen synthesis (56). Increased estrogen synthesis or exogenous estrogen treatment silences inflammation and rapidly improves the signaling function of abundant fatty tissue in obese patients (57).

## Synthetic Estrogens are Endocrine Disruptors Inhibiting the Unliganded Activation of ERs and Causing ER Deregulation

From the early 1940s, synthetic estrogens DES and EE in high doses were introduced as promising treatments for patients with advanced breast cancer (58). High doses of synthetic estrogens resulted in low rates of tumor response (<30%), while their side effects reflected serious cardiovascular and gastrointestinal toxicity as well as frequently life threatening clotting disorders (59) suggesting a genome-wide deregulation of different organs. Use of high dose synthetic estrogens against breast cancer ensured the evaluation of the anticancer effects of synthetic hormones without any information concerning the effects of endogenous estrogens.

In female transgenic mice with inactivated AF2 domain of ERs, DES treatment could not induce an estrogen-like uterotrophic response through the activation of AF1 domain (60). This experiment justified that DES may block the unliganded activation of AF1 domain, while provoking a compensatory liganded activation of the AF2 of ERs, thus mimicking an estrogen-like effect at the expense of ER deregulation (47). DES may be regarded as endocrine disruptor compound instead of an estrogenic one, and the higher the applied dose, the more serious is its toxicity.

In pregnant rats, exposure to excessive DES and EE induced mammary tumor development in both mothers and their offsprings. These synthetic estrogens equally provoked persistent alterations in the expression of estrogen regulated genes, in DNA methylation and histone modifications (61). These observations underline that even the chemically modified steroidal EE is an endocrine disruptor instead of being a bioidentical estrogen (47). In human breast cancer therapy, unbalanced inhibition and activation of ER domains *via* high dose synthetic estrogen treatment led to controversial tumor responses and serious toxic complications *via* alterations in the expression of estrogen regulated genes.

Oral contraceptives (OCs) comprising steroidal EE were developed in the early 1960s and EE became a standard component of near all forms of contraceptive pills (7). OCs comprising low doses of EE may usually work well, however; in certain women they may induce unforeseeable arterial or venous thromboembolic complications (62). OC use is especially dangerous for women with metabolic syndrome, type-2 diabetes and hypercholesterolemia (63). Use of OCs exhibits ambiguous correlations with cancer risk depending on the specific regulatory features of affected organs (47). In OC users, overall breast cancer risk is slightly increased (64, 65), while the risk of poorly differentiated ER-negative and triple negative breast cancer (TNBC) type tumors is significantly increased among them (33, 66). These findings suggest that in women with inherited or acquired defect of liganded ER signaling, long term OC use may increase the risk of poorly differentiated breast tumors *via* an additive deregulation of ER activation (47).

In contrast, OCs comprising low doses of EE may decrease endometrial cancer (67) and ovarian cancer risks (68). In anovulatory women, artificial cycles formed by OC use strongly improve insulin resistance and sex hormone imbalances in the uterus and ovaries (69, 70). OCs may reduce endometrial and ovarian cancer risks *via* a strong compensatory upregulation of the AF2 domain of ERs.

In conclusion, EE is an endocrine disruptor compound even in low doses as it is rather a partial antagonist of ER activation instead of being an agonist. Since EE has been regarded as a bioidentical estrogen, the experienced thromboembolic events and increased breast cancer risk in OC users were mistakenly regarded as complications of elevated estrogen levels (10).

Xenoestrogens deriving from the environment have endocrine disrupting properties acting as false ligands of nuclear receptors including estrogen receptors. Deregulation of estrogen receptors lead to changes in DNA methylation and histone modification leading to genomic instability. Environmental endocrine disruptor compounds may be natural like phytoestrogens and resveratrol or synthetic substances like solvents, pesticides, cleaning products and cosmetics. Many observations are suggesting that exposure to environmental endocrine disruptors do contribute to cancer, diabetes, obesity, metabolic syndrome and infertility (71).

During the development of menopausal hormone therapy (MHT) both synthetic estrogens (EE, E2) and conjugated equine estrogens (CEEs) with natural origin were applied (5). Before 2000, the early MHT studies designated all estrogenic hormones simply as exogenous estrogens without distinction between natural and synthetic formulas (10).

Increased endometrial cancer risk was reported in postmenopausal women using miscellaneous exogenous estrogens (72). From 1985, highly conflicting reports were published concerning the cardiovascular risks (73) and benefits (74, 75) in women using various estrogen formulas. In 1998, a meta-analysis study summarized that unopposed estrogen therapy, using different synthetic and natural formulas, increases the risk for endometrial hyperplasia, endometrial cancer, arterial and venous thromboembolic complications and for breast cancer (76).

In 2002, the results of a great, prospective, placebo controlled Women’s Health Initiative (WHI) study strengthened that combined CEE plus medroxyprogesterone-acetate (MPA) treatment (PremPro, Pfizer) increased the risk of breast and colon cancer, thromboembolism, cardiovascular diseases and hip fracture (9). Following these serious experiences, there was a consequential precipitous decrease in MHT use among postmenopausal women and a thorough re-evaluation of MHT practice became necessary (5). Later, in a prospective MHT study, highly toxic effects of MPA were published as compared with other synthetic progestins (77). This finding illuminated that in the WHI study published in 2002, the MPA component of PremPro may be blamed for the catastrophic results of MHT instead of the horse urine deriving Premarin (46).

Evaluating the controversial results of MHT studies; the FDA US established several times that various approved estrogen and progestin formulations may alleviate menopausal symptoms in postmenopausal women. However, their use is associated with ambiguous “risks and benefits” concerning coronary heart disease, thromboembolism as well as for endometrial and breast cancers (5).

In 2004, the results of a further great, prospective placebo controlled WHI study showed strikingly decreased breast cancer risk after using oral CEE alone; and the risk for total cancer also was slightly reduced (78). Considering the outstanding findings on breast cancer prevention by estrogen, WHI investigators performed extended studies continuing the follow up of surviving hormone treated and control patients. Further data were published on the breast cancer risk reduction among re-examined CEE treated patients in 2011 (79), in 2012 (80), in 2013 (81) in 2015 (82) and in 2020 (83). Despite the experienced consistently decreased breast cancer morbidity and mortality in the CEE treated group, WHI investigators did not support CEE use for breast cancer risk reduction.

Critical analysis of the results of MHT studies revealed that the use of estrogens with different origin and even their combinations with synthetic progestins may explain the chaos of quite controversial clinical experiences among hormone user women (10). Premarin (CEE) treatment alone proved to be a key to successful menopausal hormone therapy decreasing all health risks of women having menopausal complaints (84).

In 2021, the results of a new WHI randomized, placebo-controlled trial were published. Conjugated equine estrogen treatment (N = 10,739) significantly reduced ER-positive, PR-negative cancers and deaths from breast cancer also were reduced by 40% (85). This finding has not been demonstrated for any other pharmacological intervention. Considering the outstanding breast cancer risk reducing capacity of Premarin, authors eventually established “here is the time for change in our breast cancer risk reduction strategies and clinical practice”.

## Antiestrogens are Chemotherapeutic Agents Targeting the Liganded Activation of ERs, Which is the Principal Means of Genomic Regulation

The pharmaceutical industry developed the first drugs for the inhibition of estrogen induced activation of ERs and they were introduced into the practice of breast cancer care. Tamoxifen was regarded as a selective estrogen receptor modulator, while letrozole worked as aromatase inhibitor reducing estrogen synthesis in breast cancer patients (86).

Considering the whole population of breast cancer patients, antiestrogen treatment could not surpass the “magic” 30% of tumor response rate, similarly to the weaknesses of other endocrine therapies; such as oophorectomy or high doses of synthetic estrogens (87). The majority of even the targeted ER-positive tumors were not responsive to the endocrine treatment showing primary resistance (13). In addition, patients showing earlier good tumor responses to antiestrogens later experienced secondary resistance leading to metastatic disease and fatal outcome.

Despite the 50 years practice and advance of antiestrogen therapy, the inhibition of liganded ER activation could not become the key to the tumor free survival of breast cancer cases. Enormous efforts have been exerted worldwide to overcome the tumor “resistance” to antiestrogen therapy, which develops near in all ER-positive breast cancer patients following long term treatment.

Systemic antiestrogen treatment with tamoxifen or aromatase inhibitor, blocks the genomic processes in both the healthy cells of patient’s body and in ER-positive tumor cells. In antiestrogen responsive patients, a compensatory ovarian hyper stimulation may be experienced (88), while tumors show clinical regression. In the background, the whole body increases systemic and mammary estrogen signal *via* higher expression of ERs and activation of estrogen synthesis. In the meantime, the blockade of liganded ER activation is a crisis even for tumors promoting the upregulation of estrogen signal and DNA stabilization leading to apoptosis. Tumor response may be regarded as a common success based on the repaired genomic regulation of both patients and their tumors (39).

In patients, non-responsive to antiestrogens, various toxic side effects are experienced suggesting a genome wide blockade of the estrogen signal in the whole body. Tumor cells remain without regulatory help, similarly, like tumor cell lines *in vitro*. In tumors, the predominance of blocked, tamoxifen-bound ERs aggravates the failure of estrogen signal and DNA repair resulting in unrestrained proliferation coupled with clinically observable tumor growth. The cancer bearing mammary tissue also suffers of deregulation and cannot put up demarcation line against the local spread of tumor. In addition, deregulated remote organs cannot counteract the colonization of arriving tumor cell groups. In conclusion, tumor growth, metastatic spread and fatal outcome are the results of uncompensated blockade of liganded ER activation (40).

Molecular events behind the response and resistance of antiestrogen treated breast cancer cells were thoroughly analyzed. Different mechanisms of endocrine resistance were suggested, such as compensatory increased expression of ER coactivators (89), and a counteractive stimulation of aromatase activity (90). Recent reports identified mutations in the ERα obtained from the recurrent tumors of AI treated patients. These mutations enable the ERα to activate without ligands and presumably auto-stimulate metastatic tumor growth (91).

In *tumor cells responsive to endocrine therapy*, the increased expression and activation of ERs were typical observations counteracting the endocrine disruptor treatment (92). When tamoxifen highly stimulates compensatory ER expression and activation it may successfully arrest tumor cell survival and proliferation (93, 94). In tamoxifen sensitive tumors, the amplification of ER alpha encoding *ESR1* gene is typically coupled with highly increased expression and activation of ERs (95, 96). Both estrogen withdrawal and tamoxifen induced ER blockade recruits the coactivators of ERs so as to increase the upregulation of estrogen signal (97–99). In ER-positive tumors, tamoxifen shock provokes increasing expression of certain microRNAs helping mRNA transcripts of ERs facilitating new protein synthesis (40). Aromatase inhibitor therapy of tumor cells induces an acquired amplification of aromatase encoding *CYP19A1* gene enhancing both enzyme expression and estrogen synthesis (100). In conclusion, in antiestrogen responsive, ER-positive tumors, the principal response to the medical blockade of AF2 domain is a compensatory increased expression and liganded activation of ERs (92).

In breast tumors non-responsive to endocrine therapy, a long term tamoxifen treatment continuously stimulates ER expression, while tumors are progressively growing (101). Breast tumors becoming tamoxifen resistant exhibit highly increased expression and activation of GFRs, both IGFRs and EGFRs, besides the abundance of ERs (93). This overwhelming GFR expression is not a survival technique of tumor cells, but rather it serves a compensatory activation of accessible ERs *via* unliganded pathway (47).

In exhaustively treated non-responsive tumors, ERs mediate activating mutation on *ERBB2* gene of GFR tyrosine kinases conferring a compensatory increased unliganded activation of nuclear ERs (102). In endocrine refractory ER-positive breast cancer, *PIK3CA* gene is frequently mutated upregulating the components of the PI3K-AKT-mTOR tyrosine kinase cascade further increasing the unliganded activation of ERs (103). In tumors, under exhaustive aromatase inhibitor treatment, the extreme estrogen loss turns the ligand binding domain (LBD) of ERs responsive to GFR signal *via* an acquired mutation on *ESR1* gene (104). This gene edition is an effort for restoring the crucial estrogen signal even in the absence of estrogen (39).

In conclusion, in antiestrogen resistant tumors, increased expression and activation of ERs, GFRs and tyrosine kinase cascades do not facilitate an increased proliferation of tumor cells but rather they serve as strong feedback signals to ERs *via* unliganded activation. Activating mutations on the genes of ERs, GFRs and tyrosine kinases serve desperate efforts for restoring estrogen signal and DNA repair capacity.

Darwinian low of evolution, namely, the selection of more fit genotype/phenotype of living organisms should be specially adapted to tumors treated with endocrine disruptors. In tumors, gene amplification or even acquired new mutations occurring under antiestrogenic shock aims the restoration of DNA stabilization and apoptotic death of tumor cells serving the survival of patient (39). In conclusion, tumor cell indirectly follows on the rule of all living organisms; survival and reproduction are above all.

How can breast tumors exhibit either response or resistance to tamoxifen therapy, when they exhibit a similar abundance of ERs?

In ER-positive, antiestrogen responsive tumors, tamoxifen treatment may provoke compensatory increased ER expression and E2 synthesis *via* upregulating the circuit of ^E2^ER-BRCA-aromatase-E2-ER signal and the associated DNA stabilization. Predominance of estrogen-bound ERs over tamoxifen-blocked ones may achieve DNA repair, apoptotic tumor cell death coupled with clinical tumor response.

In ER positive, antiestrogen resistant tumors, an exhaustive tamoxifen treatment may induce abundant expression of ERs; however, the continuous treatment may achieve a predominance of tamoxifen-bound ERs over estrogen-bound ones. ERs perceiving the blockade of estrogen signal, drive increasing expression and activation of GFRs as well, targeting the unliganded activation of ERs. However, growth factor signal is incapable of stimulating tamoxifen-blocked ERs. Without a compensatory activation of ERs, the whole genomic regulation breaks down resulting in unrestrained cell proliferation and clinically experienced tumor growth.

Unexpectedly, estrogen treatment emerged as a key to restore the response of anti-hormone resistant tumors in both male prostatic cancer cases and female breast cancer patients (105). Moreover, estrogen treatment induced apoptotic death in breast cancer cell lines resistant to either long term estrogen deprivation or tamoxifen treatment (106). Considering the strong compensatory upregulation of both ER and GFR expressions in antiestrogen resistant tumors, estrogen gains an enormous potential for inducing a strong balanced activation of ERs through both liganded and unliganded pathways.

In 2021, Italian authors described the fact that all genes required to maintain genome integrity belong to the estrogen-controlled cellular signaling network requiring an upgrade to the vision of estradiol as a carcinogenic “genotoxic hormone” (107).

## Fundamental Errors in the Principles of Current Cancer Therapy

1. Cancer cells are mistakenly regarded as enemies to be killed similarly like exogenous microbes.

In reality, cancer cells are deregulated human cells having more or less remnants of the same genomic regulation like patients have in their healthy cells (42).

2. Erroneous concepts suggest that tumor initiation may be attributed to the activation of certain altered genes and their protein products.

Conversely, the vast majority of altered, mutated genes in tumors serve spontaneous efforts for the restoration of the defect of estrogen signal and DNA replication (39).

3. Cancer cells are mistakenly regarded to be enemies escaping from the regulatory control of the whole body and developing survival techniques for their unrestrained proliferation.

In reality, cancer cells have no ambitions for survival and replication, but rather use all their preserved capacities for the upregulation of estrogen signal, helping DNA repair and self directed death (31).

4. All therapeutic efforts mistakenly inhibit the remnants of crucial regulatory mechanisms in tumor cells.

In reality, the therapeutic upregulation of estrogen signal stimulates all regulatory processes in healthy cells, while induce apoptotic death in cancer cells in a Janus-faced manner (37, 42).

5. Techniques for determining the gene expression profiles of tumors have been developed and direct correlations were supposed between altered genes and the progression of the disease.

In reality, in tumor gene expression profiles, the genetic alterations may reflect compensatory efforts for the restoration of estrogen signal and DNA integrity, while these efforts may be either successful or unsuccessful. There is no parallelism between gene alterations and tumor progression (39).

6. Current genetic therapy targets amplified and mutated genes in tumors; however, the results of these efforts are controversial in clinical practice.

Conversely, endogenous estrogen upregulates the work of the whole genomic machinery and it may induce defensive gene amplification and gene edition in tumors exposed to genotoxic therapy (39, 47, 92).

7. Drug resistance is an erroneously presumed reason responsible for the failures of genotoxic therapy of breast cancer and other solid tumors.

In reality, non-responsive tumors are not resistant to therapy, but rather they are incapable of counteracting the shock of artificial genomic destruction (18).

8. In tumors, increased expression and activation of membranous growth factor receptors and their tyrosine kinase cascades are mistakenly regarded as stimulators of tumor growth.

By contrast, increased growth factor receptor activation in tumors is a compensatory effect for upregulation of weak estrogen signal *via* unliganded pathway (39, 47).

9. Therapeutic inhibition of growth factor receptors and their gene expression is not successful in tumor therapy.

Tumors, non-responsive to GFR inhibitors are not resistant, but rather their blocked GFRs are not capable of upregulation of estrogen signal *via* unliganded pathway (47).

10. Increased endogenous estrogen concentrations are mistakenly regarded as risk factors for the development and growth of breast cancer.

In reality, high estrogen levels are physiological in pregnancy. Pathologically increased estrogen level is a compensatory reaction in patients with ER resistance. These patients show clinical signs of estrogen deficiency in spite of their increased serum estrogen levels (31, 37).

11. Thromboembolic complications and increased cancer risk in synthetic estrogen user patients strengthened the misbelief that even elevated endogenous estrogen levels may cause serious diseases.

Chemically modified synthetic hormones are endocrine disruptors causing deregulation of ERs *via* partial inhibition instead of excessive stimulation (10, 47).

12. High ER expression in breast cancer cells is mistakenly evaluated as an aggressive survival technique to be therapeutically blocked.

In reality, high ER expression in breast cancers means a promising regulatory capacity for the restoration of ER signal in an estrogen deficient milieu and forecasts good prognosis for the disease (31).

13. Tumor response to antiestrogen treatment is mistakenly attributed to a successful blockade of liganded ER activation.

In reality, antiestrogen treatment is an emergency state endangering the remnants of genomic regulation in tumors. Tumor response may be experienced when the compensatory mechanisms are appropriate for the upregulation ER activation (18, 39, 92).

14. In ER-positive tumors non-responsive to endocrine therapy, the failure is mistakenly explained by the development of resistance to treatment.

In reality, tumors non-responsive to antiestrogen therapy are not resistant. The compensatory activation of estrogen signal is weak or exhausted resulting in stagnation or growth of tumors (18, 47, 92).

## Medical Stimulation of ER Protein Expression Would Be a Promising Method for Breast Cancer Prevention and Therapy

Molecular classification of breast cancers helped to reveal that increased ER expression is associated with a high differentiation of tumors. Moreover, the higher the ER expression of tumors, the better is the prognosis of the disease. In breast cancers, there is a close association between DNA repair capacity levels and ER expression status. This finding justifies that ER-positive tumors are capable of employing complex signaling pathways through both genomic and non-genomic regulation (108). In contrast, ER-negative and TNBC type tumors are poorly differentiated and the lack of hormone receptors forecasts poor prognosis for cancer patients. ER-negative and TNBC type tumors are frequently observed in patients with estrogen resistance showing low ER expression and/or decreased liganded activation of ERs (31).

Exogenous estrogen receptor was transfected into a receptor negative breast cancer cell line and estrogen activation could decrease the invasive and metastatic potential of tumor cells (109). These experimental results suggest that the medical stimulation of ER expression in patients may be an effective therapy against both ER-negative and TNBC type tumors.

In 1989, Malone and coworkers published a landmark experiment on inducing protein production in cell cultures *via* transfecting them with liposome protected messenger ribonucleic acid (mRNA) template stimulating the expression of a foreign protein *via* translation (110). In 1990, Wolff and coworkers introduced the possibility of synthesizing mRNA in a laboratory to trigger the production of a desired protein in mouse muscle (111). These studies presented the earliest steps towards mRNA vaccine development against COVID-19 pandemic (112).

The production of a desired protein *via* mRNA technology may have great possibilities in human practice beyond vaccination. Considering that ERs are the chief regulators of all genomic processes, Malone’s mRNA technology may be an excellent method for the stimulation of appropriate ER protein production in patients. Theoretically, ESR1 mRNA treatment in patients with low ER levels or ER resistance may prevent breast cancer development by stimulation of ER production and increase in estrogen regulated gene expression. In addition, in patients with advanced ER negative and TNBC type tumors, a preoperative ESR1 mRNA treatment may achieve marked tumor regression coupled with stimulating defensive reactions in the adjacent tissues. In the postoperative phase, local ESR1 mRNA treatment improves the genomic functions in the remaining breast tissue and inhibits the development of recurrent tumor. In breast cancer cases with multiple metastatic lesions, systemic ESR1 mRNA treatment may achieve regression of tumors at all sites besides improvement of the metabolic and immunological status of patients. The dream of perfectly selective tumor therapy and the concomitant safeguarding of healthy tissues seem to be realized.

## Conclusion

The developing new trends in anticancer fight are targeting mutated genes by gene therapy and attacking their altered protein products by immunotherapy. However, the accumulation of altered genes and their abundant protein products in tumors may not show a parallelism with the progression of the disease as they are rather spontaneous compensatory efforts for DNA repair instead of oncogenic activities. All developed new methods of gene therapies are targeting the compensatory genome improving processes, while tumor cells need rather medical help supporting their DNA restoration so as to achieve apoptotic death.

Recognition of the molecular mechanisms of estrogen receptor deregulation *via* synthetic estrogen and antiestrogen treatment, helped to reveal the omnipotent curing capacity of endogenous estrogens *via* balanced liganded and unliganded activation of ERs.

## Footnotes

1 Rosetta Stone - Wikipedia. Available at: https://en.wikipedia.org/wiki/Rosetta_Stone (Accessed October 9, 2014).
